# Male-Specific Transfer and Fine Scale Spatial Differences of Newly Identified Cuticular Hydrocarbons and Triacylglycerides in a *Drosophila* Species Pair

**DOI:** 10.1371/journal.pone.0016898

**Published:** 2011-02-14

**Authors:** Joanne Y. Yew, Klaus Dreisewerd, Cássia Cardoso de Oliveira, William J. Etges

**Affiliations:** 1 Temasek Life Sciences Laboratory and Department of Biological Sciences, National University of Singapore, Singapore, Singapore; 2 Institute of Medical Physics and Biophysics, University of Münster, Münster, Germany; 3 Program in Ecology and Evolutionary Biology, Department of Biological Sciences, University of Arkansas, Fayetteville, Arkansas, United States of America; AgroParisTech, France

## Abstract

We analyzed epicuticular hydrocarbon variation in geographically isolated populations of *D. mojavensis* cultured on different rearing substrates and a sibling species, *D. arizonae*, with ultraviolet laser desorption/ionization mass spectrometry (UV-LDI MS). Different body parts, i.e. legs, proboscis, and abdomens, of both species showed qualitatively similar hydrocarbon profiles consisting mainly of long-chain monoenes, dienes, trienes, and tetraenes. However, *D. arizonae* had higher amounts of most hydrocarbons than *D. mojavensis* and females of both species exhibited greater hydrocarbon amounts than males. Hydrocarbon profiles of *D. mojavensis* populations were significantly influenced by sex and rearing substrates, and differed between body parts. Lab food–reared flies had lower amounts of most hydrocarbons than flies reared on fermenting cactus substrates. We discovered 48 male- and species-specific hydrocarbons ranging in size from C_22_ to C_50_ in the male anogenital region of both species, most not described before. These included several oxygen-containing hydrocarbons in addition to high intensity signals corresponding to putative triacylglycerides, amounts of which were influenced by larval rearing substrates. Some of these compounds were transferred to female cuticles in high amounts during copulation. This is the first study showing that triacylglycerides may be a separate class of courtship-related signaling molecules in drosophilids. This study also extends the kind and number of epicuticular hydrocarbons in these species and emphasizes the role of larval ecology in influencing amounts of these compounds, many of which mediate courtship success within and between species.

## Introduction

Exchange of chemical, auditory, and visual cues during courtship in many species is required for successful courtship and mating. Species and population-specific signaling is often required by both sexes prior to fertilization in multiply mating species where mate choice decisions may result in increased fitness for offspring due to sexual selection. In different species or more diverged populations, these signals can relay information about species status and influence sexual isolation [1], [2]. Perhaps the best-studied chemical cues in animals are epicuticular hydrocarbons (CHCs) in *Drosophila* that serve as contact pheromones during physical contact phases of courtship. Gustatory receptors on male foretarsi bristles and labial palps (or proboscis) are responsible for recognizing female low volatility pheromones [3], [4] expressed on the abdomen and genital regions [5]. Bristles and sensillae in and around the female terminalia including the vaginal plate, the eighth tergite, and anal plates [6] and perhaps the ventral abdomen are possible sites for male CHC recognition during courtship, but this issue has yet to be resolved. Hydrocarbon “perfuming” or rub-off experiments have demonstrated the pheromonal role of CHCs as either species or population specific compounds that influence mating success in different *Drosophila* species [7], [8], [9], [10]. Some CHCs attract potential mates while others are known to have a repellent effect [11], [12], [13]. Further, some compounds transferred during copulation, primarily from males to females, are deposited on the female anogenital cuticle that can inhibit remating by other males [14], [15], [16], [17].

Until recently, most CHC analysis was performed with gas chromatography-mass spectrometry (GC-MS) where most non-polar CHCs were recovered using brief, whole-fly hexane washes. Some workers also used sequential elutions of CHC extracts over silver nitrate impregnated silica gel beads to separate groups of alkanes, alkenes, and alkadienes using successive epicuticle washes of hexane, 2% ether in hexane, and 25% ether in hexane [18], [19], [20]. Unsaturated CHCs were derivatized with dimethyl disulfide, and the resulting thiomethyl derivatives were analyzed by GC-MS to identify double bond positions [21]. More polar epicuticular compounds were excluded using these protocols, and so most conclusions concerning the identification of other classes of lipids and CHCs and their roles in courtship success have been restricted to nonpolar fractions. Longer wash periods and more polar solvents resulted in CHCs from deeper in the cuticle and smaller lipids and triacylglycerides from internal sources that are not likely to be involved in pheromone recognition (E. Toolson, personal communication).

Several MS-based methods for CHC analysis have been recently introduced that complement GC-MS. Direct Analysis in Real-Time (DART) MS uses a helium plasma to desorb and ionize CHCs prior to MS analysis. The CHC samples are collected with a fine metal probe touching different regions of the fly body and subsequently placed in the plasma stream of the instrument. This method provides a finer scale spatial resolution of CHC expression compared to whole animal extraction and was previously used to show CHC composition differences between various parts of single, live flies. However, DART MS does not reveal double bond positions in unsaturated molecules and cannot differentiate between linear and branched compounds [22]. Everaerts et al. [23] employed solid phase micro-extraction (SPME) with GC-MS. As with DART-MS, sample preparation does not require killing the flies, thus allowing repeated sampling of CHCs under different experimental conditions. Matrix- assisted laser desorption/ionization (MALDI) time-of-flight (TOF) mass spectrometry with a lithium or sodium 2,5-dihydroxybenzoate matrix has been used to chemically image fly wings [24]. Analysis of extracts with the lithiated matrix provided coverage comparable to GC-MS [25]. Electrospray ionization (ESI) MS can also be used to detect oxygen-containing hydrocarbons from extracts [14]. However, when cuticular extracts are used, spatial information is lost and the insects must be sacrificed. In addition, extracts may require pre-fractionation in order to reduce sample complexity. Ultraviolet laser desorption/ionization mass spectrometry (UV-LDI-o-TOF MS) uses a UV laser to desorb and ionize compounds directly from the cuticles of intact flies. The 200 µm laser beam diameter provides improved spatial resolution compared to the previous methods; however, the vacuum conditions necessary for analysis are usually lethal for the animal. This method has been used with individual intact flies and has revealed large numbers of new cuticular compounds including some oxygenated fractions, but unlike GC-MS, does not detect alkanes [14]. Of these techniques, GC-MS is best suited for structural elucidation. Thus, current understanding of the numbers, kinds, function, and genetic basis of these compounds is rapidly changing due to fine scale detection of a largely undetected spectrum of compounds in the insect epicuticle using these techniques.

Here, we reassessed epicuticular CHC variation in a pair of cactophilic drosophilids, *D. mojavensis* and *D. arizonae*, because CHCs in these species have been shown to vary geographically and are influenced by preadult rearing conditions [19], [26], [27]. We suspected that UV-LDI-o-TOF MS would reveal additional CHC components in addition to those already identified with GC-MS and provide a new look at how different rearing substrates might influence body part-specific differences in CHC profiles involved in courtship signaling. In *D. mojavensis*, epicuticular CHCs serve as contact pheromones that mediate sexual isolation between geographically isolated populations [9], [28], [29], and species-specific CHC differences have been described [19]. Courtship and mating in both species occurs around naturally occurring cactus “rots” in small groups of flies. Males approach females from behind and initiate courtship with a stereotyped, population-specific wing vibration or courtship song [30], [31], followed by repeated proboscis extensions to “taste” the female's genitalia. If a female has not recently mated, the male continues courting if the female remains stationary and “drums” his foretarsi on the female's ventral abdomen while continuing proboscis extensions. Female acceptance is signaled by wing spreading, thereby allowing the male to mount and copulate; otherwise, females move or fly away at this stage of courtship [32].

### Natural history of *D. arizonae* and *D. mojavensis*

Members of the large *D. repleta* group [33], *D. mojavensis* and *D. arizonae* are restricted to the cactus deserts and arid lands of North America [34], [35], [36]. Both species share a common ancestor, are considered sibling species, and together with the more ancestral *D. navojoa*, form the *D. mojavensis* cluster [33], [36]. The range of *D. arizonae* extends from Arizona, USA to Guatemala, and overlaps with that of *D. mojavensis* in southern Arizona, and Sonora, Sinaloa, and southern Baja California, Mexico. The ecology and biogeography of *D. mojavensis* have been extensively studied [37], [38], [39] where peninsular Baja California populations carry out their life cycles in pitaya agria cactus, *Stenocereus gummosus*, and mainland Mexico populations use organ pipe cactus, *S. thurberi*, with occasional use of sina cactus, *S. alamosensis*, with which it sometimes shares with *D. arizonae*. In the Mojave Desert, *D. mojavensis* uses California barrel cactus, *Ferocactus cylindraceous*, and on Santa Catalina Island near Los Angeles, CA, *Opuntia* spp. are used for feeding and breeding. Host use in *D. arizonae* is far broader, but usually associated with species of columnar cacti, including use of fermenting cactus fruits [40]. Baja California populations of *D. arizonae* are recent, associated with a tendency for *D. arizonae* to be commensals with humans. In the present study, we focus on Baja California and mainland Sonora populations of *D. mojavensis*, and a sympatric Sonoran population of *D. arizonae*.

### Epicuticular hydrocarbons of *D. mojavensis* and *D. arizonae*

Previous gas chromatography-mass spectrometry analysis of hexane-extracted CHCs of both species revealed approximately 30 different branched alkanes, alkenes, branched alkenes, and alkadienes, with the most abundant components having odd numbered carbon chains ranging in size from C_29_ to C_39_ [19], [20], [26]. Most quantitative variation described was due to differences between species, sex, populations, and rearing substrates where C_35_ alkadienes accounted for close to half of the total CHCs per fly [26]. Two major peaks for these species, 2-methyloctacosane and 2-methyltricontane, methylalkanes with chain lengths of C_29_ and C_31_, are not considered in the present study because UV-LDI-o-TOF mass spectrometry does not detect alkanes and cannot differentiate between branched and linear compounds [14].

Studies of rearing substrate effects on cuticular hydrocarbon profiles of *D. arizonae* and *D. mojavensis* [19], [20] demonstrated significant differences between cactus and lab food reared flies, so subsequent studies have employed cactus-reared flies only [9], [26], [29], [41]. Host rearing effects on agria vs. organ pipe cactus influenced a small number of hydrocarbon components in Baja California and mainland populations, but these differences due to cactus species were far smaller than those for cactus vs. lab food. We show that cactus-reared flies using UV-LDI-o-TOF MS analysis revealed quantitatively similar CHC profiles as shown by previous GC-MS analyses, but uncovered previously undetected oxygenated CHC components, as well a large number of different CHCs and putative triacylglycerides localized in the anogenital region of males. Some of these compounds were transferred to females during copulation.

## Materials and Methods

### Fly husbandry

Populations of *D. mojavensis* and *D. arizonae* were collected in nature, returned to the lab, and cultured on banana food [42] until the experiments began. A population of *D. mojavensis* from Punta Prieta, Baja California originated from 456 wild-caught adults in January 2008, and a mainland population from Las Bocas, Sonora was started with 1264 wild adults collected in March 2009. A population of *D. arizonae* also from Las Bocas, Sonora was initiated with 446 wild-caught adults.

Initially we reared *D. mojavensis* and *D. arizonae* on lab food in half pint bottles to characterize CHC variation, and then we compared two populations of *D. mojavensis* reared on lab food and both agria and organ pipe cactus. All fly cultures were reared in an incubator programmed at 27°C during the day and 17°C at night on a 14:10 LD cycle. Cactus cultures were set up in plugged half pint bottles with 75 g of aquarium gravel at the bottom covered with a 5.5-cm-diameter piece of filter paper. Bottles were then autoclaved, and after 60 g of either agria or organ pipe tissues were in place, autoclaved again for 10 min. After cooling to room temperature, each culture was inoculated with 0.5 ml of a pectolytic bacterium, *Erwinia cacticida* [43] and 1.0 ml of a mixture of seven yeast species common in natural agria and organ pipe rots [44]: *Dipodascus starmeri*, *Candida sonorensis*, *Starmera amethionina*, *Candida valida*, *Pichia cactophila*, *Pichia mexicana* and *Sporopachydermia cereana*. Eggs were collected from aged adults for 10 hr and washed in deionized water, 70% ethanol, and again in sterile deionized water. Eggs were counted out in groups of 200, transferred to a 1 cm^2^ piece of sterilized filter paper, and placed on fermenting cactus. All emerged adults were collected daily from each culture, separated by sex, and housed in small groups in shell vials on banana food in the incubator described above until sexually mature (12–14 days).

### Preparation of flies for ultraviolet laser desorption ionization mass spectrometry (UV-LDI MS) analysis

Individual flies were anesthetized and mounted with fine forceps onto adhesive tape (G304, Plano, Wetzlar, Germany) attached to a glass cover slip. The cover slip was attached to a custom-built sample plate with adhesive tabs. To prevent potential cross-contamination, separate forceps were used for male and for female flies. Up to 12 flies were typically placed on the sample plate at once. The integrity of the fly body remained intact during analysis in the mass spectrometer. We assessed CHC differences between forelegs, proboscis, and ventral abdomens of Las Bocas *D. mojavensis* and *D. arizonae* males and females reared on laboratory media. One foreleg from each adult was assayed. For the rearing substrates study, CHC profiles from the forelegs, proboscis, and ventral abdomens of male and female *D. mojavensis* that had been reared to eclosion on fermenting agria or organ pipe cactus tissues vs. those that had been reared on laboratory media were also compared. Flies from Baja California and mainland populations were compared.

The anogenital regions of males of both species were characterized, as were the anogenital regions of females before and after copulation in order to detect CHC transfer after mating. The anogenital and ventral abdomen regions of males from the rearing substrates study were also compared for a more extensive set of CHCs and putative triacylglycerides not found elsewhere on the flies (see below) in order to determine whether amounts of these compounds were influenced by rearing substrates and population differences.

### Laser desorption/ionization orthogonal time-of-flight mass spectrometry

This mass spectrometer was described in Dreisewerd et al. [45] and is equipped with an N_2_ laser emitting 3 ns long pulses at a wavelength of 337 nm with a repetition rate of 30 Hz. The laser beam spot size on a sample is ca 200 µm in diameter and has a flattop intensity profile. Ions are generated in a buffer gas environment using 2 mbar of argon gas. The elevated pressure was found to enhance the detection of hydrocarbons. For acquisition of mass spectra, 900 laser pulses were applied over 30 sec. Laser fluence (light energy per pulse and area) was adjusted to values moderately above the ion detection threshold, corresponding to values between 100–200 J/m^2^. The position of the sample plate was adjusted in 10 µm steps during measurements in order to optimize signal intensity. Overall signal intensity can vary from sample to sample due to individual biological variation as well as the position of the fly on the sample plate. Mass resolution (full width at half maximum) was about 10,000, sufficient to distinguish between two neighboring hydrocarbon species differing in mass by about 50 mDa. Mass accuracy was about 20–30 ppm throughout all measurements. All LDI MS data were acquired in positive ion mode. Mass spectra were processed using the MoverZ software (v. 2001.02.13, Genomic Solutions, Ann Arbor, MI). Potassiated molecules formed the dominant peaks for signals corresponding to hydrocarbons in all recorded LDI mass spectra. The signal intensity for each CHC is defined as the area under the monoisotopic peak of the potassiated ion species, as calculated by MoverZ software. Elemental composition assignments are based on the assumption that the observed and theoretical mass values agree within +/−0.02 Da and that the neutral CHC molecules contain only C, H, and O atoms (thus neglecting the unlikely occurrence of N and S).

### Electrospray ionization (ESI) MS

Cuticular extracts from adult *D. arizonae* and *D. mojavensis* were prepared by placing 30 males in a 2∶1 chloroform: methanol (v/v) solution for 20 min at room temperature. Extracts were evaporated with a gentle stream of N_2_ and dissolved in chloroform/methanol/ether with or without 1 mM ammonium acetate prior to analysis. Two different ESI MS instruments were used to analyze the extracts and to perform collision-induced dissociation (CID) for partial structural characterization of putative triacylglycerides: 1) a quadrupole time-of-flight (QTOF) mass spectrometer (Waters/Micromass) and 2) a LTQ Orbitrap hybrid mass spectrometer (Thermo Scientific).

### Data analysis

Signal intensities for groups of hydrocarbons were compared across species, populations, sexes, different regions of the fly body, different culture media, and between virgin females and those that had recently mated. The latter comparison was qualitative as most transferred compounds were male-specific. For all multivariate analyses, we first calculated logcontrasts of the proportions of each hydrocarbon of the total signal intensities to eliminate multicollinearity among sample peak amounts if simple proportions had been used [46]. Because there is no internal standard when using UV-LDI mass spectrometry, this data transformation is necessary in order to carry out multivariate statistical analysis [7]. We chose a large, reproducible sodium adduct peak present in each sample, C_35:2Na_, as the divisor except in the anogenital region study where we used the C_35:2_ peak. Each logcontrast was calculated as,
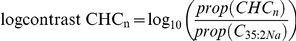



We carried out multivariate analysis of variance (MANOVA) to assess logcontrasts of CHC profiles from different parts of the flies and differences due to sex, species, and larval diets. Principal Components Analysis (PCA) was performed to identify sources of covariation among CHCs and ANOVAs were carried out to interpret which treatment effects influenced variation in PC scores. Canonical Discriminant Function (CDF) Analysis was performed to help visualize differences between treatment effects of interest. All statistical analyses were performed using SAS [47].

## Results

### CHC identification

**Figure 1:**
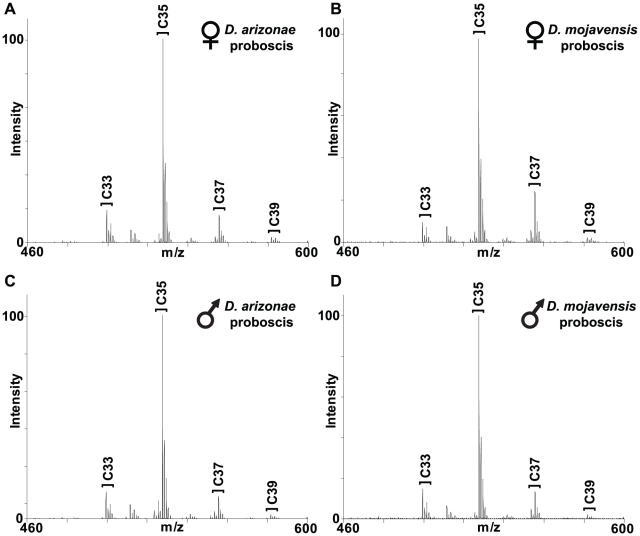
UV-LDI-o-TOF mass spectra from *D. arizonae* and *D. mojavensis* females and males for proboscis data only. The MS analyses show variation in the abundance of major groups of CHCs sampled from the adult female (A, B) and male (C, D) proboscis. Each labeled cluster contains hydrocarbons with 33 – 39 carbon atoms in length bearing 1–4 double bonds (see Table 1).

Analysis by UV-LDI MS identified 15 CHCs that were present on leg, proboscis, and ventral abdomen epicuticles of lab food-reared male and female *D. mojavensis* and *D. arizonae* (Figure 1, Table 1). The compounds were detected as intact molecules with a cation adduct and assignments of CHC elemental composition were made on the basis of high accuracy measurement of the mass to charge ratio. Most of the signals corresponded to monoene and diene CHCs and were consistent with previous GC-MS results [26], except for some trienes and the tetraenes that were not detected before. Here, signal intensity of individual CHC components is dependent on concentration as well as chemical composition. Differences in signal intensity thus indicate relative quantitative differences and not absolute amounts. As with earlier studies, the most abundant signals in the CHC profile corresponded to C_31_–C_35_ dienes and monoenes. A C_30:0H_ component was present in small amounts and rarely observed in GC-MS analyses [19], [26], but we have included it here. In contrast to GC-MS analysis, no signals corresponding to alkanes were detected using UV-LDI MS. Previous GC-MS analysis showed that there are two identified branched alkanes (2-methyloctacosane and 2-methyltricontane) and one minor component (11-and 13-methyldotricontane). These comprise ca 15% of total CHCs of cactus-reared flies based on GC-MS analysis [26]. In addition, linear and branched compounds cannot be differentiated based on mass alone. One known CHC species, C_34:2_, could not be reliably detected due to the presence of overlapping isotopic signals from another CHC components.

**Table 1:** Elemental composition of hydrocarbons detected by direct UV-LDI-o-TOF mass spectrometry in the cuticle of the forelegs, proboscis, and ventral abdomens of male and female *Drosophila mojavensis* and *D. arizonae*.

Hydrocarbon chainlength and structure	Elemental composition	Calculated Massof [M+K]+ Ion
C30:0 OH	C_30_ H_62_ O	477.44
C33:3 - tritricontatriene	C_33_ H_62_	497.47
C33:2 - tritricontadiene	C_33_ H_64_	499.469
C33:1 - methyldotricontene	C_33_ H_66_	501.49
C35:4 - pentatricontatetraene	C_35_ H_64_	523.483
C35:3 - pentatricontatriene	C_35_ H_66_	525.498
C35:2 - pentatricontadiene	C_35_ H_68_	527.496
C36:2	C_36_ H_70_	541.51
C36:1	C_36_ H_72_	543.53
C37:4 - heptatricontatetraene	C_37_ H_68_	551.492
C37:3 - heptatricontatriene	C_37_ H_70_	553.518
C37:2 - heptatricontadiene	C_37_ H_72_	555.534
C39:4	C_39_ H_72_	579.52
C39:3	C_39_ H_74_	581.545
C39:2	C_39_ H_76_	583.56

### CHC variation in D. arizonae and D. mojavensis

**Figure 2:**
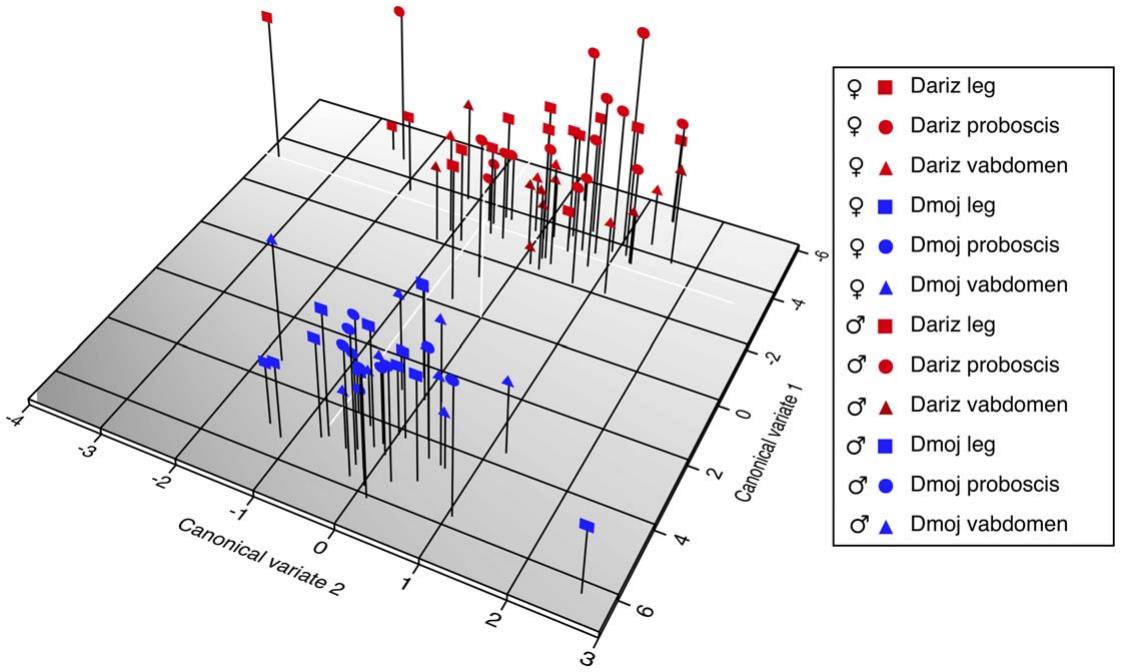
Canonical Discriminant Function plot of male and female body part CHC variation in lab food reared *D. mojavensis* (Dmoj) and *D. arizonae* (Dariz). Vabdomen  =  ventral abdomen. Species and sex differences were observed in lab food-reared flies, but no significant differences in CHC expression were found when comparing body parts within the same species. Observations are different individuals for each body part.

There were significant differences in CHC profiles between *D. mojavensis* and *D. arizonae* as well as large sex differences as shown by MANOVA, but a significant Sex X Species interaction (Table 2) made species differences more apparent than differences due to gender (Figure 1). Canonical discriminant function analysis was used to plot group differences along different axes of CHC covariation that clearly showed these species differences (Figure 2). Sex differences contributed mostly to variation in CV 2 (Figure 2). Significant differences in CHC composition were also observed between the legs, proboscis, and ventral abdomen using MANOVA, but this source of variation was much smaller than either species or sex differences in CHCs (Table 2).

**Table 2:** MANOVA results for differences in amounts of the 15 major hydrocarbon components in lab food-reared male and female *D. arizonae* and *D. mojavensis* assessed from different body parts, i.e. legs, proboscis, and ventral abdomen.

Source of variation	Wilks' λ	F	df	P
Sex	0.3052	8.35	15,55	<0.0001
Species	0.1030	31.94	15,55	<0.0001
Body part	0.4757	1.65	30,110	0.033
Sex*species	0.3845	5.87	15,55	<0.0001
Sex*part	0.6431	0.91	30,110	0.610
Species*part	0.6918	0.74	30,110	0.812
Sex*species*part	0.7013	0.71	30,112	0.853

Principal components analysis (PCA) was also employed to analyze relative differences in signal intensity between *D. arizonae* and *D. mojavensis*, and characterized different covarying groups of CHCs. The first PC accounted for 67 percent of the variation in this data set with PC 7 accounting for less than one percent of the total variation, so we restricted our focus on the first 6 PCs (Table 3). All CHCs positively covaried with PC 1 including higher level of C_33:3_ (10-, 12- & 14-tritricontatriene), C_33:2_ (8,24-tritricontadiene and 7,25-tritricontadiene), C_35:3_, and C_35:2_ (9,25-pentatricontadiene, 8,26-pentatricontadiene, and 7,27-pentatricontadiene) in *D. arizonae* than *D. mojavensis*, consistent with Etges and Jackson [26] with one exception (Table 3B). Levels of C_33:3_ were greater in *D. mojavensis* than *D. arizonae* in that study, but this may have resulted from either population level variation or because all flies were reared on fermenting cactus in Etges and Jackson (2001) as opposed to the present analysis where cultures of *D. mojavensis* and *D. arizonae* that were reared on lab food. Females had significantly greater amounts of C_33:1_, C_35:3_, C_37:4_, C_37:3_, C_37:2_, C_39:4_, C_39:3_, and C_39:2_ than males (Table 3B), also consistent with some of the differences in Etges and Jackson [26], but the latter C_39_ components were not segregated into different peaks in that study. PC 3 was most influenced by variation in C_33:3_, C_37:4_, and C_37:3_, where PC 4 – 6 were characterized by higher loadings for smaller groups of different CHCs.

**Table 3A:** The first six Principal Components showing covariation among 15 cuticular hydrocarbons from the legs, proboscis, and ventral abdomens of male and female *D. mojavensis* and *D. arizonae*, and B, significant differences between species and sexes for each HC revealed by posthoc comparisons of least square means for each hydrocarbon component.

A. Hydrocarbon	PC 1	PC 2	PC 3	PC 4	PC 5	PC 6
C30:OH	0.276	0.072	−0.220	−0.120	−0.088	0.276
C33:3 - tritricontatriene	0.113	0.471	0.678	0.431	0.099	0.113
C33:2 - tritricontadiene	0.290	0.187	−0.050	0.008	−0.207	0.290
C33:1 - methyldotricontene	0.246	0.199	−0.200	−0.262	0.203	0.246
C35:4 - pentatricontatetraene	0.267	0.184	0.143	−0.246	−0.306	0.267
C35:3 - pentatricontatriene	0.299	0.069	0.072	0.035	−0.229	0.299
C35:2 - pentatricontadiene	0.278	−0.126	−0.125	0.315	−0.302	0.278
C36:2	0.285	0.123	−0.012	0.011	−0.094	0.285
C36:1	0.258	0.101	−0.165	−0.181	−0.254	0.258
C37:4 - heptatricontatetraene	0.237	−0.357	0.430	−0.352	0.010	0.237
C37:3 - heptatricontatriene	0.220	−0.502	0.377	−0.190	0.102	0.220
C37:2 - heptatricontadiene	0.263	−0.349	−0.143	0.320	0.212	0.263
C39:4	0.211	0.313	−0.039	−0.319	0.619	0.211
C39:3	0.290	−0.126	−0.112	0.322	0.127	0.290
C39:2	0.279	−0.052	−0.139	0.263	0.365	0.279
Eigenvalue	10.007	1.291	0.917	0.670	0.639	0.375
Proportion of total variance	0.667	0.086	0.061	0.045	0.043	0.025

B. Hydrocarbon	PC 1	Species	Sex			
C30:OH	0.276	ariz > moj *				
C33:3	0.113	ariz > moj ****				
C33:2	0.290	ariz > moj ****				
C33:1	0.246	ariz > moj ***	F>M **			
C35:4	0.267	ariz > moj ****				
C35:3	0.299	ariz > moj ****	F>M *			
C35:2	0.278	ariz > moj *				
C36:2	0.285	ariz > moj ****				
C36:1	0.258	ariz > moj *				
C37:4	0.237	ariz > moj *	F>M *			
C37:3	0.220		F>M *			
C37:2	0.263		F>M ****			
C39:4	0.211	ariz > moj ***	F>M *			
C39:3	0.290		F>M **			
C39:2	0.279		F>M ****			

*P<0.05,

**P<0.01,

***P<0.001,

****P<0.0001.

In order to examine factors contributing to the differences found between the two species, ANOVAs were performed on each of these 6 PCs. The analysis revealed that PC 1 was influenced by sex and species differences, but PC 2 variation was caused by species differences and a Sex x Species interaction (Table S1). The remaining PCs were also influenced by these sources of variation in different ways, but body part-specific variation was not significant for any PC consistent with the MANOVA (Table 2, Table S1). Thus, most of the CHC variation detected here in lab food-reared *D. arizonae* and *D. mojavensis* by UV-LDI-o-TOF mass spectrometry was due to species and sex differences, and not variation between body parts.

### Rearing substrate effects on *D. mojavensis* CHC profiles

Both populations of *D. mojavensis* reared on lab food and both host cacti showed significant CHC differences for all main effects and interactions in a MANOVA (Table S2). The largest sources of CHC variation were due to Population, Sex, Food, and the Sex x Population interaction. These differences were consistent with the known geographic, sex, and rearing substrate effects on adult CHCs, including Sex x Geographic Region interactions. Here, this was manifested in the significant Sex x Population interaction term (Table S2) consistent with the region specific sex differences in CHC profiles [9]. Similar to the PC results for the two species reared on lab food, this population comparison based on the same 15 CHC components resulted in six PCs that each represented more than five percent of the total variation (Table 4). Loadings on PC 1 were all positive, with +/− loadings on the other PCs similar to those for lab food-reared *D. arizonae* and *D. mojavensis* in (Table 3).

**Table 4A:** The first six Principal Components showing covariation among 15 cuticular hydrocarbons from the legs, proboscis, and ventral abdomens of male and female *D. mojavensis* reared on different larval substrates from two populations.

A. Hydrocarbon	PC 1	PC 2	PC 3	PC 4	PC 5	PC 6
C30:OH	0.256	0.064	−0.116	−0.450	0.101	−0.156
C33:3 - tritricontatriene	0.147	0.665	0.250	0.217	0.059	0.108
C33:2 - tritricontadiene	0.180	0.628	0.028	−0.009	0.080	−0.223
C33:1 - methyldotricontene	0.285	−0.071	−0.207	−0.192	−0.160	−0.170
C35:4 - pentatricontatetraene	0.262	−0.052	−0.205	0.330	−0.039	0.207
C35:3 - pentatricontatriene	0.306	0.037	0.018	0.046	−0.027	0.092
C35:2 - pentatricontadiene	0.274	−0.079	−0.300	−0.193	0.317	−0.339
C36:2	0.302	−0.020	−0.077	−0.013	0.325	−0.017
C36:1	0.205	−0.043	−0.517	0.498	0.149	0.291
C37:4 - heptatricontatetraene	0.207	−0.139	0.385	−0.303	0.424	0.631
C37:3 - heptatricontatriene	0.316	−0.041	0.034	−0.070	−0.256	0.001
C37:2 - heptatricontadiene	0.296	−0.098	0.118	−0.023	0.030	−0.058
C39:4	0.163	−0.313	0.504	0.468	0.284	−0.477
C39:3	0.298	−0.072	0.169	0.008	−0.451	0.065
C39:2	0.289	−0.084	0.168	0.023	−0.441	0.046
Eigenvalue	8.684	1.446	0.957	0.771	0.685	0.561
Proportion of total variance	0.579	0.096	0.064	0.051	0.046	0.037

B. Hydrocarbon	Population 1	Sex	Food 2	Body part 3		
C30:OH	LB > PP ****	ns	LF<OP, AG **	ns		
C33:3	LB > PP ****	F>M **	LF<OP, AG ****	LG, PB>VAB ****		
C33:2	LB > PP ****	ns	LF<OP, AG ***	LG, PB>VAB ****		
C33:1	LB > PP ****	ns	LF>OP, AG *	PB>LG, VAB ****		
C35:4	LB > PP ****	ns	ns	LG, PB>VAB *		
C35:3	LB > PP ****	F > M *	LF<OP<AG ****	ns		
C35:2	ns	ns	ns	PB>VAB, LG **		
C36:2	ns	ns	LF<OP, AG ****	PB>VAB, LG ***		
C36:1	LB > PP *	ns	OP>LF, AG **	PB>LG>VAB ***		
C37:4	LB > PP ***	F>M ***	ns	PB, VAB>LG ***		
C37:3	LB > PP ****	F>M *	AG>LF, OP *	ns		
C37:2	LB > PP ****	F>M ****	LF<OP, AG ***	PB>LG, VAB ***		
C39:4	LB > PP *	F>M ****	ns	PB>LG, VAB **		
C39:3	LB > PP ****	F>M **	ns	PB>LG, VAB **		
C39:2	LB > PP ****	F>M ****	AG<OP, LF **	PB>LG, VAB ***		

B. Significant differences between sexes, populations, body parts, and substrates for each CHC revealed by post hoc comparisons of CHC least square means for PC 1.

1 LB  =  Las Bocas, Sonora, and PP  =  Punta Prieta, Baja California.

2 LF  =  lab food, OP  =  organ pipe cactus, AG  =  agria cactus.

3 LG  =  leg, PB  =  proboscis, VAB  =  ventral abdomen.

*P<0.05,

**P<0.01,

***P<0.001,

****P<0.0001.

Comparisons of least square means for each CHC component revealed that the mainland, Las Bocas population had greater amounts of all 15 CHCs except the C_35_ and C_36_ alkadienes than the Punta Prieta, Baja California population (Table 4B). Variation due to sex was similar to that observed between species, described above, where females had greater amounts of the C_37_ and C_39_ CHCs than males. Rearing substrates influenced 10 of these CHCs: six of these differences were caused by significantly lower CHC amounts in lab food vs. cactus-reared flies (Table 4B). For two other CHCs, C_36_ monoenes and C_37_ trienes, lab food caused reduced CHC amounts equivalent to levels caused by one of the cactus species, and in only two instances did lab food cause increased CHC amounts. A general pattern seen here, first reported in Stennett and Etges [19], is that cactus-reared flies tend to have more CHCs than lab food reared flies. Most often, agria and organ pipe-reared flies did not differ for most CHCs, but Sex x Cactus interactions were more common where rearing on organ pipe cactus decreased CHC amounts in males and increased them in females as compared to agria-reared flies [9].

**Figure 3:**
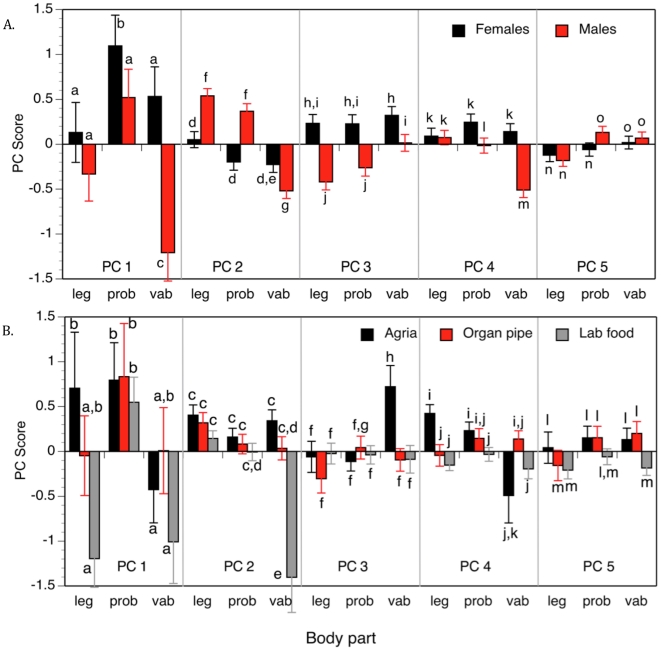
Principle Component scores for CHC covariation on *D. mojavensis* body parts. Principle component analysis of legs, proboscis, (prob), and ventral abdomens (vab) revealed differences in CHC expression between (A) males and females, and (B) due to rearing substrates.

Differences in CHC amounts found on distinct body parts were significant for 12/15 CHCs, where in a majority of cases, proboscis CHC amounts were significantly higher than leg or ventral abdomen amounts (Table 4B, Figure 3). For all 15 CHCs, amounts were almost always lowest in the ventral abdomen region, particularly in males. In contrast, leg CHC amounts were equivalent to those on the proboscis for the C_33:2_, C_33:3_, and C_35:4_ components (Table 4B). As before, we performed ANOVAs of the first six Principal Components and found significant effects of population, sex, rearing substrates, and body parts for PC 1, as well as a significant population by sex interaction (Table S3). Variation in PC 2, with the high positive loadings of C_33:2_ and C_33:3_, and negative loading of C_39:4_ (Table 4), was influenced by almost every factor in the ANOVA, as was PC 4, similar to the MANOVA results (Table S2). Thus, nearly all of the CHCs covarying in different ways, i.e. PC 1-6, varied in *D. mojavensis* due to population, sex, rearing substrates, and their interactions.

Overall CHC differences indicated by PC 1 scores (Table 4, S3), showed that female proboscis CHCs were more abundant than on other body parts, and greater than male proboscis amounts in most cases (Figure 3A). The significance of body part differences in CHC amounts may be inferred in the context of courtship behaviors and CHC perception by both sexes. A significant Sex X Body part interaction was expected if CHC amounts differed between males and females consistent with the exchange of chemical signaling during courtship, but this interaction was complicated by population differences as shown by the Population x Sex x Body part interaction for PC 1 (Table S3). Male proboscis extension contacting female genitalia, “licking”, and then male foretarsi “drumming” of the female ventral abdomen are the main physical contact signals prior to copulation [32], so female perception of male CHC profiles should be facilitated if CHC amounts are higher on the male proboscis and forelegs (Figure 3A). Male PC 1 and PC 2 scores for legs and proboscis were significantly greater than for the ventral abdomen region (Figure 3A), consistent with male pheromone signaling with proboscis and leg CHCs [5]. A contrasting pattern was observed for PC 3 indicating different covarying groups of these CHCs may serve as male mating signals detected by females in direct contact with males during this phase of courtship. PC 4 scores were very similar to PC 1 scores (Figure 3A), PC 5 variation due to sex was not significant, and variation due to body part differences was significant for all but PC 6 (Table S3).

Rearing substrates also significantly influenced CHC variation in these populations of *D. mojavensis*, both as a main effect for PC 1–5 and as a Food x Body part interaction for PC 2–4 (Table S3). Rearing substrates were involved in several other higher order interactions, but we were mainly interested in how these substrates influenced CHC expression on different body parts. Rearing substrates had little effect on proboscis CHCs for any of the PCs, yet lab food caused significant lowering of CHC amounts on the ventral abdomen for PC 1 and 2 and on legs for PC 1 (Figure 3B). Agria cactus caused increased CHC levels on legs vs. organ pipe cactus and lab food, but tended to decrease CHC amounts on the ventral abdomen as lab food did for PC 1. These rearing substrate effects were quite similar to those caused by sex differences in CHCs between body parts (Figure 3A) suggesting that the low CHC levels on male ventral abdomens for PC 1 and 2 were significantly influenced by lab food. Overall, these rearing substrate effects expressed on different body parts underscore the complexity of CHC expression and the difficulties in trying to understand CHC mediated mate choice using artificial laboratory substrate cultured *D. mojavensis* [cf. 48].

### Variation and male transfer of anogenital region CHCs

Forty-eight cuticular hydrocarbons and at least 15 other lipid compounds ranging in size from C_22_ to C_50_ were consistently detected in mass spectra acquired from the anogenital region of *D. mojavensis* and *D. arizonae* males (Table 5). Only eight of these CHCs were observed on other parts of the fly except for adjacent regions of the ventral abdomen (see below). A number of other low intensity signals were found inconsistently and are not included in this study.

**Table 5A:** Observed cuticular hydrocarbons and putative triacylglycerides (TG) in the anogenital regions of male *D. mojavensis* and *D. arizonae* detected by direct UV-LDI-o-TOF mass spectrometry, C_22_ to C_34_.

A. Hydrocarbon chainlength and double bond number^1^	Elemental composition	Calculated Massof [M+K]^+^ Ion	Relative intensity*D. mojavensis* 2	Relative intensity*D. arizonae* 2
C22:1	C_22_H_42_O_2_	377.28 *	+	n/d
C24:1	C_24_H_46_O_2_	405.31 *	+	n/d
C26:2	C_26_H_48_O_2_	431.33 *	+	n/d
C26:1	C_26_H_50_O_2_	433.34 *	++	n/d
C28:2	C_28_H_52_O	443.37 *	+	+
C28:1	C_28_H_54_O†	445.38 *	+	++
C28:2	C_28_H_52_O_2_ †	459.36 *	++	n/d
C28:1	C_28_H_54_O_2_	461.38 *	++	n/d
Unknown	-	463.38 *	+	+
C31:2 - hentricontadiene	C_31_H_60_	471.43 *	+	+
C31:1	C_30_H_58_O	473.41 *	+	++
TG	C_25_H_42_O_6_	477.26	+	++
C30:2	C_30_H_56_O_2_ †	487.39 *	++++	+
TG	C_26_H_44_O_6_	491.28 *	+	+
TG	C_26_H_46_O_6_	493.29 *	+	+
TG	C_26_H_48_O_6_	495.31	n/d	+
C33:2 - tritricontadiene	C_33_H_64_	499.46 *	++	++
C33:1 - hentriacontene	C_33_H_66_	501.48 *	++	++
C34:2 - tetratricontadiene	C_34_H_66_	513.48	+	++
C32:2	C_32_H_60_O_2_ †	515.42 *	++++	+

B. Hydrocarbon chainlength and double bond number^1^	Elemental composition	Calculated Massof [M+K]^+^ Ion	Relative intensity*D. mojavensis* 2	Relative intensity*D. arizonae* 2
TG	C_28_H_48_O_6_ †	519.31 *	+++	++
TG	C_28_H_50_O_6_	521.32 *	+	+
C35:3 - pentatricontatriene	C_35_H_66_	525.48 *	+	+^○^+
C35:2 - pentatricontadiene	C_35_H_68_	527.50 *	++++	++++
TG	C_29_H_48_O_6_ †	531.31 *	++	+++
TG	C_29_H_50_O_6_ †	533.32 *	+	+++
TG	C_30_H_52_O_6_	547.34 *	+	+
C37:3 - heptatricontatriene	C_37_H_70_	553.51 *	+	+
C37:2 - heptatricontadiene	C_37_H_72_	555.53 *	++	+^○^+
TG	C_31_H_50_O_6_	557.32	+	+
TG	C_31_H_52_O_6_ †	559.34 *	+++	++++
TG	C_31_H_54_O_6_	561.36 *	++	++
Unknown	-	577.34 *	+	+
TG	C_33_H_56_O_6_	587.37	+	+
TG	C_33_H_58_O_6_	589.39	n/d	+
C44:3	C_44_H_82_O_2_ †	681.60	+	++
C44:2	C_44_H_84_O_2_ †	683.61	+	++
C46:3 hexatetracontatriene	C_46_H_86_O	693.63	n/d	+
C46:4	C_46_H_84_O_2_	707.61	n/d	+
C46:3	C_46_H_86_O_2_ †	709.63	++	++
C46:2	C_46_H_88_O_2_	711.64	+	++
TG	C_42_H_74_O_6_	713.51	+	n/d
TG	C_42_H_76_O_6_	715.53	+	+
C48:4	C_48_H_88_O_2_	735.64	n/d	+
C48:3	C_48_H_90_O_2_ †	737.66	+	++
Unknown	-	741.54	+	+
C50:4	C_50_H_92_O_2_	763.67	n/d	+
C50:3	C_50_H_94_O_2_	765.69	n/d	++

B. *IBID*, anogenital region cuticular hydrocarbons and TGs detected, C_35_ to C_50_.

1 Proposed chemical compositions are listed as the number of carbon atoms followed by the number of double bonds in the hydrocarbon chain; TG: putative triacylglyceride, with preliminary structure supported by electrospray mass spectrometry.

2 The relative abundance of each CHC species is calculated by dividing the area under the monoisotopic peak by the total area of all CH peaks detected in the same experiment: ++++, >10% of the total area; +++, 5%–10%; ++,1%–5%; +, <1%; n/d: not detected.

*The 29 CHCs used in the statistical analyses of *D. mojavensis* involving the anogenital and ventral abdomen regions.

† Compound transferred to females during copulation, see Fig. 5.

Most of the non-CHC lipids specific to the anogenital region likely correspond to triacylglycerides based on exact mass measurements and chemical composition assignments. For each of the putative triacylglycerides, the predicted number of oxygen atoms (6) and degree of unsaturation was consistent with those found in typical triacylglyceride molecules. In addition, the putative triacylglycerides molecules appeared as clusters of peaks separated by 28.03, indicating elongation by C_2_H_4_ groups, another typical feature of triacylglyceride structure.

**Figure 4:**
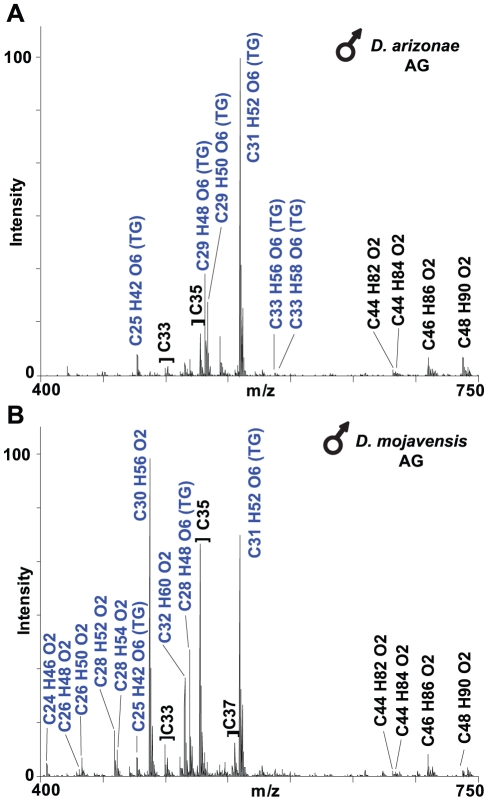
Representative mass spectra from male *D. arizonae* (A) and *D. mojavensis* (B). UV-LDI MS analysis reveals profile differences in CHCs and putative triacylglycerides (TG) that are specific to the anogenital region (AG). Each compound is labeled with the predicted elemental composition. Compounds found only in the AG are labeled in blue.

To obtain additional structural data, we fragmented protonated C_31_H_52_O_6_ and C_29_H_48_O_6_, two of the major compounds found in the anogenital region of *D. arizonae*, by ESI tandem MS. The data acquired were consistent with triacylglycerides containing one singly unsaturated C_16_H_30_O_2_ and C_18_H_34_O_2_ fatty acid chain, respectively, at one position of the glycerol backbone and possibly two identical C_5_H_8_O_2_ residues at the other two positions (data not shown). Additional ESI tandem MS analysis of other putative triacylglycerides suggested that these compounds fragmented in a similar way, thus indicating a similar chemical structure. It must be emphasized that tandem MS data are not unequivocal proof of the overall structures, and that further chemical analysis will be needed to confirm this preliminary assignment and exact chemical structures of the compounds. There were clear qualitative and quantitative differences in amounts of these compounds (Figure 4), but we did not statistically analyze these differences between *D. arizonae* and *D. mojavensis* males. Most of these compounds have not been observed before, and with the exception of the non-oxygen containing hydrocarbons, most were not found on females.

**Figure 5:**
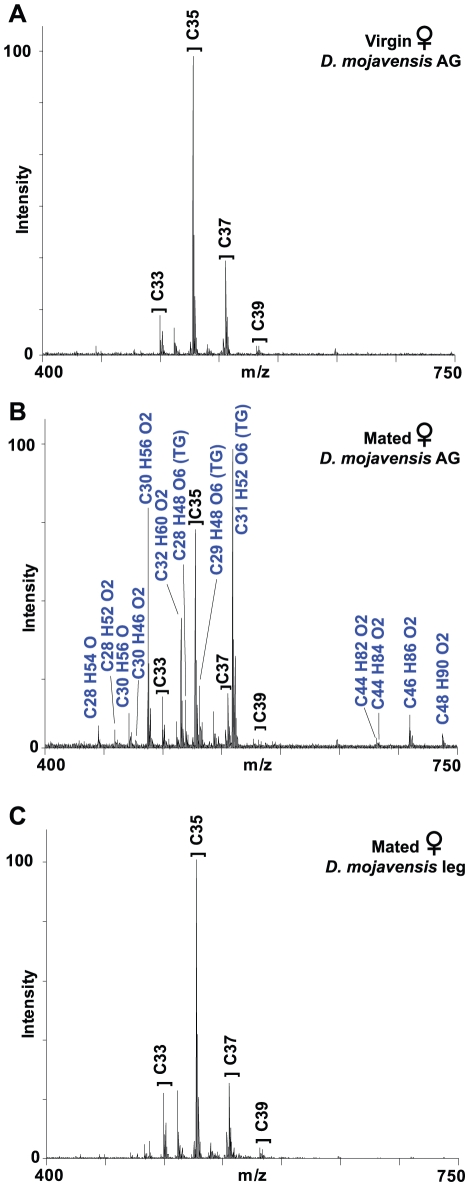
Transfer of male *D. mojavensis* compounds to females during mating. UV-LDI mass spectra of female *D. mojavensis* before (A) and after mating (B, C) show that 13 CHCs and triacylglycerides (TG), specific to the male anogenital region, are transferred to the female anogenital (AG) region but not the legs (B, C). Male-specific AG compounds are labeled in blue. See Table 5 for CHC and triglyceride designations.

**Figure 6:**
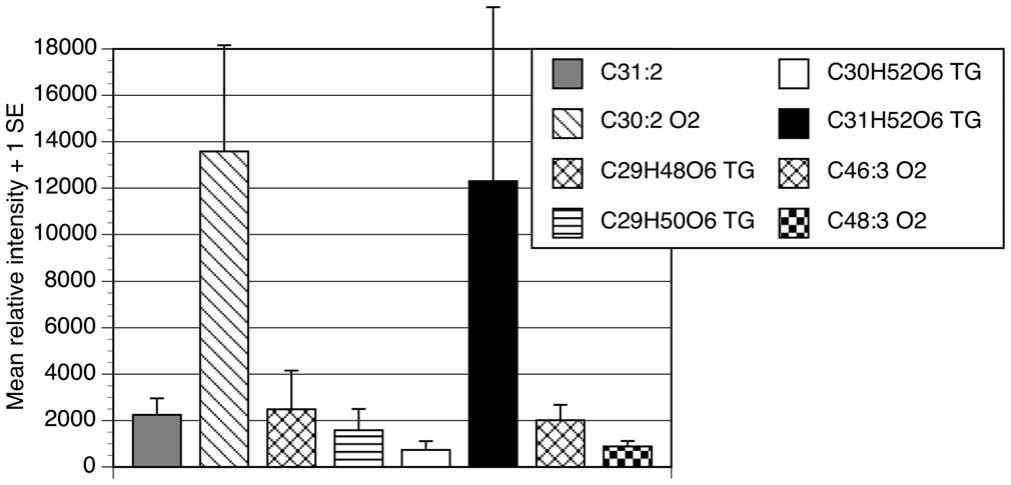
Relative amounts of male-specific compounds transferred from male *D. mojavensis* to females during copulation. Only the compounds with expression specific to the male anogenital region are analyzed. The 8 CHCs and putative triacylglycerides used in this analysis were reliably detected on all mated females (n = 3); however, up to twelve male-specific compounds could be found on female cuticles 24 h after mating.

Male specific anogenital CHCs and putative triacylglycerides were transferred to females during copulation (Figures 5, 6). We included hentricontadiene, C_31:2_, for comparison because it was found in virgin females in very low quantities near levels of background noise (data not shown), but mated females had up to 100X as much (Figure 6). We detected transfer of 12 CHCs and putative triacylglycerides after copulation (Figure 5, Table 5) with significant amounts of seven CHC components including four putative triacylglycerides and C_46_ and C_48_ hydrocarbons, both of the latter containing O_2_ groups.

**Figure 7:**
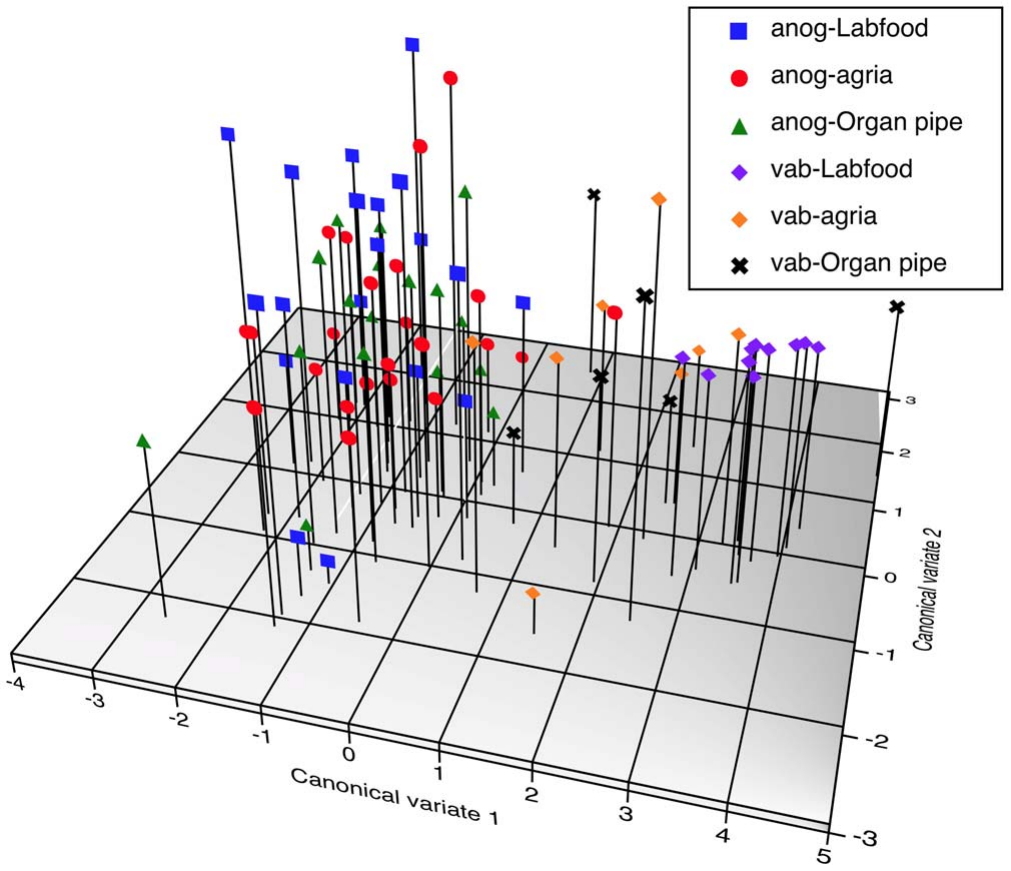
Canonical Discriminant Function plot showing the effects of larval diet on adult CHC expression. Male anogenital (anog) and ventral abdomen (vab) CHC variation in *D. mojavensis* vary depending on whether the larvae were reared on lab food, agria cactus, and organ pipe cactus.

Many of these anogenital CHCs and putative triacylglycerides were also found in the adjacent ventral abdomen area in males of both populations of *D. mojavensis*. Amounts of 28 of these compounds that we could reliably detect (see Table 5) significantly differed between populations, rearing substrates, and between the ventral abdomen and anogenital regions (Table 6). However, many ventral abdomen CHCs of lab food-reared Las Bocas males were nearly undetectable, and we could not detect most of these 28 CHCs and putative triacylglycerides on the abdomens of organ pipe cactus-reared males from Punta Prieta, so these latter males were not included in this analysis. These qualitative differences undoubtedly caused the significant Food x Part interaction, but precluded estimation of a Population x Food x Body part interaction (Table 6). Canonical discriminant function analysis of these 28 male specific CHCs revealed that anogenital and ventral abdomen regions were clearly differentiated along the first canonical variate (Figure 7) indicating significant CHC differences between these two regions (Wilk's 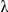 = 0.254, F = 5.87, df = 28,56, P<0.0001), and by differences in preadult diet (Wilk's 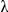
_ = _0.257, F = 1.91, df = 56,110, P = 0.002). As before, anogenital and ventral abdomen CHCs of lab food-reared flies were significantly different from those of agria and organ pipe cactus-reared flies (P<0.0001 and P = 0.002, respectively), but there were no quantitative or qualitative CHC profile differences between agria and organ pipe cactus-reared *D. mojavensis* (P = 0.853). Thus, rearing substrates also influenced variation in anogenital region specific CHCs and those in the adjacent ventral abdomen area, and thus some of the CHCs and putative triacylglycerides that were transferred to females during copulation.

**Table 6:** MANOVA results for differences in the 28 hydrocarbon and putative triacylglyceride components assessed by UV-LDI MS from the ventral abdomen and anogenital regions of male *D. mojavensis* reared on three larval diets; lab food, agria cactus, and organ pipe cactus.

Source	Wilks' λ	F Value	df	Pr>F
Population	0.3831	2.76	28,48	0.001
Food	0.0199	10.42	56,96	<0.0001
Body part	0.0915	17.02	28,48	<0.0001
Population X Food	0.2691	1.59	56,96	0.023
Population X Part	0.6378	0.97	28,48	0.52
Food X Part	0.0176	11.19	56,96	<0.0001

See text for details.

## Discussion

Chemical signaling systems in *D. arizonae* and *D. mojavensis* are far more complex than previously thought with the discovery of a large spectrum of CHCs and putative triacylglycerides that were specific to the anogenital and surrounding ventral abdomen regions of males. UV-LDI-o-TOF MS verified previous GC-MS findings with whole fly CHC extracts [20], [26] and identified several previously undescribed alkatrienes. Alkatetraenes, linear hydrocarbons with 4 double bonds were, until now, unknown in *Drosophila* (though a number of these compounds have been reported for *Lepidoptera* [49]). A few pheromonal components, e.g. C_34_ alkadienes [41] were not reliably measured in this study because they overlapped with isotopic signals from other compounds. Since alkanes are currently not detected with this method, variation in the major CHC components 2-methyloctacosane and 2-methyltricontane could not be assessed. This is a notable difficulty in understanding the roles of covarying groups of hydrocarbons as pheromones because these two CHCs were positively associated with male mating success [see Table 4 in 29] and it would have been useful to know if these two CHCs are also spatially differentiated on adult body parts.

Few other *Drosophila* systems have lent themselves to in-depth analysis of how preadult rearing environments, including natural breeding sites, cause adult CHC variation. In *D. mojavensis*, different host cacti influenced CHC variation that in turn determined male mating success both within and between populations [28; Havens et al., unpublished data,29,41]. Here, when flies were reared on lab food, both *D. arizonae* and *D. mojavensis* showed qualitatively similar CHC profiles, but *D. arizonae* tended to have more of each type of CHC than *D. mojavensis*, and females usually had higher CHC amounts than males. These sex differences were consistent with past studies [19], [26], but species differences in CHC amounts were not always comparable because the these studies used different populations reared on other cactus species and not always lab food.

Rearing substrate, sex, and population effects on CHC profiles in geographically isolated populations of *D. mojavensis* revealed by UV-LDI-o-TOF MS also confirmed most previous results. Analysis of 5 mainland and 6 Baja California populations of *D. mojavensis*, including the two analyzed here, reared on agria and organ pipe cactus revealed females had higher amounts of most CHCs than males, as well being larger than males [P<0.0001; see Table 5 in 9]. However, while thorax sizes of mainland populations tend to be larger than those from Baja California, they were not significantly so (P = 0.055). There were also many CHCs that showed Sex x Geographical Region interactions indicating that Baja California and mainland populations are characterized by alternate male – female hydrocarbon cues, consistent with the present study (Table S2). In comparison with lab food, agria and organ pipe cactus also caused increased CHC amounts (Figure 3), suggesting that adult CHC precursors are more easily extracted and synthesized from fermenting cactus tissues than lab media, the former containing well characterized communities of cactophilic bacteria and yeasts required for cactus tissue fermentation [50], [51]. Previous experiments have shown that autoclaved cactus tissues not inoculated with yeasts or bacteria preclude larval development [52], [53]. Investigations of the interdependence of cactus, yeasts, and *Drosophila* have revealed complex interactions between cactus tissue chemistry, bacteria and yeast physiology, and the resulting fermentation by-products on the fitness of the drosophilids using various species of cacti [54]. Some studies have even shown optimal foraging by larvae for particular yeast species in nature and preference for these yeasts in laboratory tests [55]. Since these fermenting cactus substrates also directly influence courtship behavior by reducing premating isolation between Baja and mainland populations of *D. mojavensis*, the reductions in CHC amounts on male legs and ventral abdomens in lab food reared flies (Figure 3) suggest that this lab food effect may be expressed by males during the “drumming” phase of courtship.

These cactus effects also are relevant to interspecific sexual isolation because *D. arizonae* and *D. mojavensis* exhibit higher sexual isolation when reared on cactus substrates than lab food [56]. Host plant sharing by *D. arizonae* and *D. mojavensis* in nature is not widespread [38], [40], [57], so the role of cactus-induced shifts in CHC composition in these species should be evaluated on a host specific basis. Although CHCs have not yet been directly implicated in sexual isolation between these two species, there is some evidence for CHC differences where populations are sympatric [26]. In other sympatric species of *Drosophila*, changes in CHCs have been shown to contribute to reproductive isolation [8], [58]. So far, for desert species of *Drosophila*, only male courtship songs have been implicated in interspecific sexual isolation where different song types are recognized by females in a species-specific manner [31].

Species and sex-specific CHC variation in drosophilids can be both quantitative and qualitative. In some species, male or female specific CHCs have provided some of the best examples of a pheromonal role for these compounds [reviewed in 59,60]. Variation in the most abundant CHCs in *D. mojavensis* and *D. arizonae* is quantitative, with no known species or sex specific hydrocarbons except for those in the male anogenital region. One small C_33:2_ peak in these two species differs qualitatively from their closest relative, *D. navojoa* [26], in which it is a major CHC suggesting increased CHC differentiation exists in more distantly related *D. repleta* group species. Body part-specific CHC variation revealed by UV-LDI-o-TOF MS also included rearing substrate-specific spatial differences in CHC abundance. The 28 CHCs and putative triacylglycerides (Tables 5, 6) on ventral abdomens of lab food-reared *D. mojavensis* males from Las Bocas was undetectable, and most of these 28 compounds were found in very small amounts on the abdomens of organ pipe cactus-reared males from Punta Prieta. Since most of the compounds were easily detectable in the anogenital region, we assume their presence on the ventral abdomen is due to physical translocation by male preening. Why they were absent or in much reduced amounts only in these two cases is unknown.

Chemical differences in the anogenital region were often species-specific where amounts of 14/48 male CHCs and putative triacylglycerides were detectable in only one of these species (Table 5). These qualitative differences in anogenital CHCs and putative triacylglycerides are strongly suggestive of a chemically based species-specific signaling system, but we have yet to implicate a functional role for any of these compounds. This is the first study to show triacylglyceride-like compounds may serve as a separate class of courtship-related signaling molecules in drosophilids. In a similar study with *D. melanogaster*, physical transfer of male, anogenital specific CH503, identified as (3*S*, 11*Z*, 19*Z*)- 3-acetoxy-11,19-octacosadien-1-ol [61], caused inhibition of female remating for at least 10 days [14]. Further analyses of these compounds in *D. mojavensis* and *D. arizonae* are needed, as well as estimates of how long these male-derived chemicals remain on the female cuticle and which tissues or glands contribute to their synthesis. It seems that a comprehensive understanding of CHC variation and function in *Drosophila* will require analysis of a far wider range of compounds than previously considered.

## Supporting Information

Table S1 **ANOVA results for the first six cuticular hydrocarbon Principal Components from male and female** ***D. mojavensis*** **and** ***D. arizonae*** **from legs, proboscis, or ventral abdomen detected by direct UV-LDI-o-TOF mass spectrometry.**
(DOC)

Table S2 **MANOVA results for the 15 hydrocarbon components assessed by UV-LDI MS from legs, proboscis, and ventral abdomens of male and female** ***D. mojavensis*** **reared on three larval diets; lab food, agria cactus, and organ pipe cactus.** See text for details.
(DOC)

Table S3 **ANOVA results for the first six cuticular hydrocarbon Principal Components based on the 15 CHCs (** **Table 4** **) from male and female** ***D. mojavensis*** **reared on lab food and two cactus substrates from legs, proboscis, or ventral abdomen detected by direct UV-LDI-o-TOF mass spectrometry.**
(DOC)
