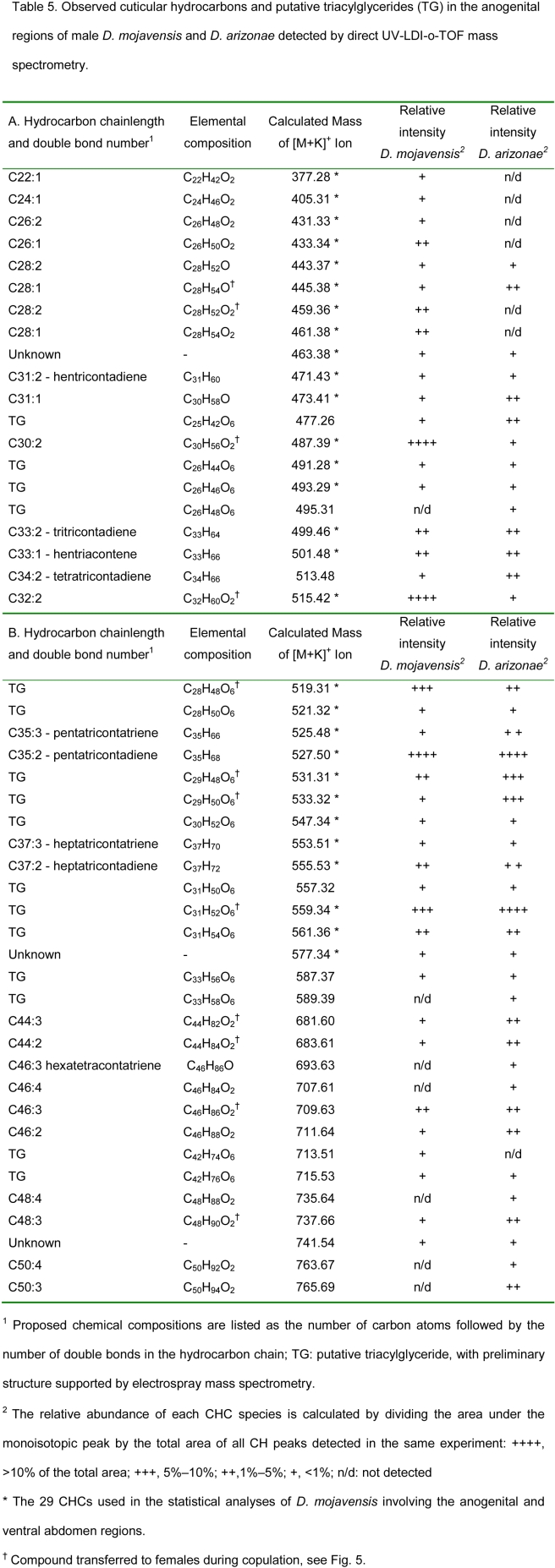# Correction: Male-Specific Transfer and Fine Scale Spatial Differences of Newly Identified Cuticular Hydrocarbons and Triacylglycerides in a *Drosophila* Species Pair

**DOI:** 10.1371/annotation/39235c4d-3231-4539-bbeb-85c0295cd62b

**Published:** 2011-09-15

**Authors:** Joanne Y. Yew, Klaus Dreisewerd, Cássia Cardoso de Oliveira, William J. Etges

Table 5 is missing section A. The complete Table 5 can be viewed here: 

**Figure:**