# Supplementary material for: Male-Specific Transfer and Fine Scale Spatial Differences of Newly Identified Cuticular Hydrocarbons and Triacylglycerides in a Drosophila Species Pair
Source: PLoS One. 2011 Feb 14;6(2):e16898. doi: 10.1371/journal.pone.0016898 (PMC3038915; doi:10.1371/journal.pone.0016898)
Supplement: Table S1 — ANOVA results for the first six cuticular hydrocarbon Principal Components from male and female D. mojavensis and D. arizonae from legs, proboscis, or ventral abdomen detected by direct UV-LDI-o-TOF mass spectrometry. (DOC) [file pone.0016898.s001.doc]

Supplemental Table 1. ANOVA results for the first six cuticular hydrocarbon Principal Components from male and female *D. mojavensis* and *D. arizonae* from legs, proboscis, or ventral abdomen detected by direct UV-LDI-o-TOF mass spectrometry.

|  |  | PC 1 | | PC 2 | | PC 3 | | PC 4 | | PC 5 | | PC 6 | |
| --- | --- | --- | --- | --- | --- | --- | --- | --- | --- | --- | --- | --- | --- |
| Effect | df | F | Pr | F | Pr | F | Pr | F | Pr | F | Pr | F | Pr |
| Sex | 1/80 | 9.17 | 0.004 | 3.02 | ns | 0.0 | ns | 8.54 | 0.005 | 29.00 | < 0.0001 | 1.01 | ns |
| Species | 1/80 | 12.26 | 0.001 | 26.08 | < 0.0001 | 9.22 | 0.003 | 2.67 | ns | 5.60 | 0.021 | 0.10 | ns |
| Body part | 2/80 | 1.13 | ns | 0.73 | ns | 2.01 | ns | 0.12 | ns | 1.76 | ns | 2.18 | ns |
| Sex*species | 1/80 | 1.61 | ns | 5.63 | 0.020 | 0.48 | ns | 1.60 | ns | 10.26 | 0.002 | 1.09 | ns |
| Sex*part | 2/80 | 0.59 | ns | 0.43 | ns | 1.24 | ns | 0.82 | ns | 1.21 | ns | 1.77 | ns |
| Species*part | 2/80 | 0.51 | ns | 0.56 | ns | 1.03 | ns | 0.88 | ns | 0.09 | ns | 0.25 | ns |
| Sex*species*part | 2/80 | 1.16 | ns | 0.10 | ns | 1.56 | ns | 0.43 | ns | 0.74 | ns | 1.05 | ns |
