# Supplementary material for: Male-Specific Transfer and Fine Scale Spatial Differences of Newly Identified Cuticular Hydrocarbons and Triacylglycerides in a Drosophila Species Pair
Source: PLoS One. 2011 Feb 14;6(2):e16898. doi: 10.1371/journal.pone.0016898 (PMC3038915; doi:10.1371/journal.pone.0016898)
Supplement: Table S2 — MANOVA results for the 15 hydrocarbon components assessed by UV-LDI MS from legs, proboscis, and ventral abdomens of male and female D. mojavensis reared on three larval diets; lab food, agria cactus, and organ pipe cactus. See text for details. (DOC) [file pone.0016898.s002.doc]

Supplemental Table 2. MANOVA results for the 15 hydrocarbon components assessed by UV-LDI MS from legs, proboscis, and ventral abdomens of male and female *D. mojavensis* reared on three larval diets; lab food, agria cactus, and organ pipe cactus. See text for details.

| Source | Wilks' λ | F Value | df | Pr > F |
| --- | --- | --- | --- | --- |
| Population | 0.1045 | 182.82 | 15,320 | < 0.0001 |
| Sex | 0.3347 | 42.41 | 15,320 | < 0.0001 |
| Food | 0.3019 | 17.49 | 30,640 | < 0.0001 |
| Body part | 0.5169 | 8.34 | 30,640 | < 0.0001 |
| Population X Sex | 0.5464 | 17.71 | 15,320 | < 0.0001 |
| Population X Food | 0.5044 | 8.70 | 30,640 | < 0.0001 |
| Population X Part | 0.6308 | 5.53 | 30,640 | < 0.0001 |
| Sex X Food | 0.4950 | 8.99 | 30,640 | < 0.0001 |
| Sex X Part | 0.7047 | 4.08 | 30,640 | < 0.0001 |
| Food X Part | 0.4921 | 4.15 | 60,1251.3 | < 0.0001 |
| Pop X Sex X Food | 0.6454 | 5.22 | 30,640 | < 0.0001 |
| Pop X Sex X Part | 0.7054 | 4.07 | 30,640 | < 0.0001 |
| Sex X Food X Part | 0.4138 | 5.29 | 60,1251.3 | < 0.0001 |
| Pop X Sex X Food X Part | 0.4746 | 4.39 | 60,1251.3 | < 0.0001 |
