# Supplementary material for: Male-Specific Transfer and Fine Scale Spatial Differences of Newly Identified Cuticular Hydrocarbons and Triacylglycerides in a Drosophila Species Pair
Source: PLoS One. 2011 Feb 14;6(2):e16898. doi: 10.1371/journal.pone.0016898 (PMC3038915; doi:10.1371/journal.pone.0016898)
Supplement: Table S3 — ANOVA results for the first six cuticular hydrocarbon Principal Components based on the 15 CHCs ( Table 4 ) from male and female D. mojavensis reared on lab food and two cactus substrates from legs, proboscis, or ventral abdomen detected by direct UV-LDI-o-TOF mass spectrometry. (DOC) [file pone.0016898.s003.doc]

|  |  | PC 1 | | PC 2 | | PC 3 | | PC 4 | | PC 5 | | PC 6 | |
| --- | --- | --- | --- | --- | --- | --- | --- | --- | --- | --- | --- | --- | --- |
| Effect | df | F | Pr | F | Pr | F | Pr | F | Pr | F | Pr | F | Pr |
| Population | 1/334 | 77.34 | < 0.0001 | 0.69 | ns | 20.28 | < 0.0001 | 7.02 | 0.009 | 435.87 | < 0.0001 | 0.95 | ns |
| Sex | 1/334 | 12.32 | 0.0005 | 12.87 | 0.0004 | 38.07 | < 0.0001 | 19.28 | < 0.0001 | 1.12 | ns | 2.09 | ns |
| Population*Sex | 1/334 | 5.69 | 0.018 | 8.31 | 0.0042 | 76.31 | < 0.0001 | 39.95 | < 0.0001 | 0.58 | ns | 1.67 | ns |
| Food | 2/334 | 3.23 | 0.041 | 36.62 | < 0.0001 | 4.60 | 0.011 | 4.1 | 0.017 | 11.94 | < 0.0001 | 1.11 | ns |
| Population*Food | 2/334 | 2.66 | ns | 13.99 | < 0.0001 | 14.39 | < 0.0001 | 5.33 | 0.005 | 11.2 | < 0.0001 | 5.17 | 0.006 |
| Sex*Food | 2/334 | 2.90 | ns | 46.44 | < 0.0001 | 7.50 | 0.0006 | 4.18 | 0.016 | 3.42 | 0.034 | 5.07 | 0.007 |
| Pop*Sex*Food | 2/334 | 2.62 | ns | 21.26 | < 0.0001 | 9.71 | <.0001 | 4.19 | 0.016 | 1.85 | ns | 1.71 | ns |
| Body part | 2/334 | 6.86 | 0.001 | 31.57 | < 0.0001 | 4.01 | 0.019 | 7.25 | 0.001 | 5.15 | 0.006 | 1.06 | ns |
| Population*Body part | 2/334 | 1.72 | ns | 27.63 | < 0.0001 | 0.81 | ns | 18.55 | < 0.0001 | 1.36 | ns | 4.74 | 0.009 |
| Sex*Body part | 2/334 | 2.40 | ns | 15.05 | < 0.0001 | 1.66 | ns | 7.01 | 0.001 | 1.63 | ns | 1.76 | ns |
| Pop*Sex*Body part | 2/334 | 4.26 | 0.015 | 18.26 | < 0.0001 | 3.67 | 0.026 | 7.61 | 0.001 | 7.55 | 0.0006 | 0.85 | ns |
| Food*Body part | 4/334 | 1.44 | ns | 24.27 | < 0.0001 | 5.73 | 0.0002 | 7.08 | < 0.0001 | 1.05 | ns | 1.83 | ns |
| Pop*Food*Body part | 4/334 | 1.42 | ns | 24.71 | < 0.0001 | 5.62 | 0.0002 | 9.7 | < 0.0001 | 1.08 | ns | 0.41 | ns |
| Sex*Food*Body part | 4/334 | 0.46 | ns | 34.84 | < 0.0001 | 5.11 | 0.0005 | 6.62 | < 0.0001 | 1.03 | ns | 2.3 | ns |
| Pop*Sex*Food*Part | 4/334 | 2.07 | ns | 22.04 | < 0.0001 | 5.83 | 0.0002 | 6.84 | < 0.0001 | 1.34 | ns | 0.67 | ns |

Supplemental Table 3. ANOVA results for the first six cuticular hydrocarbon Principal Components based on the 15 CHCs (Table 4) from male and female *D. mojavensis* reared on lab food and two cactus substrates from legs, proboscis, or ventral abdomen detected by direct UV-LDI-o-TOF mass spectrometry.
